# Carbapenem-resistant *Klebsiella pneumoniae* infection causing rupture of graft artery in solid organ recipients: Case report and review of literature

**DOI:** 10.1097/MD.0000000000017878

**Published:** 2019-11-11

**Authors:** Yun-Shi Cai, Heng Xiao, Shu Zhang, Mao Li, Si-Min Liang, Zheng-Rong Shi, Cheng-You Du

**Affiliations:** a Department of Liver Surgery and Liver Transplantation; b Department of Urology and Renal Transplantation, The First Affiliated Hospital of Chongqing Medical University, Chongqing, P.R. China.

**Keywords:** carbapenem-resistant *Klebsiella pneumoniae*, graft artery, rupture, solid organ transplantation

## Abstract

**Rationale:**

Donor-derived bacterial infection is a rare cause of morbidity after solid organ transplantation (SOT) but associated with significant morbidity and mortality, deaths caused by carbapenem-resistant *Klebsiella pneumoniae* (CRKP) infection account for a considerable proportion of postoperation mortality rate in liver and kidney recipients. The arterial rupture as a result of fungal arteritis is occasionally described, while the rupture of graft vascular anastomosis after SOT due to donor-derived CRKP infection is rarely reported.

**Patients concerns:**

We reported 1 patient with donor-derived CRKP infection following liver transplantation and 2 patients following renal transplantation (1 liver and 2 kidneys were from the same donor), who experienced sudden abdominal pain and abdominal hemorrhage almost at the same time after organ transplantation.

**Diagnosis:**

The patients were diagnosed as graft arteries rupture due to corrosion caused by CRKP infection based on computed tomography scan, blood culture, laparotomy, and pulse-field gel electrophoresis.

**Interventions:**

Anti-shock treatment, exploratory laparotomy, broad-spectrum antibiotics, and abdominal puncture and drainage were given.

**Outcomes:**

The liver recipient survived as well as the liver graft, still under treatment of multiple abdominal infections. The 2 renal recipients were alive after resection of the renal grafts and underwent hemodialysis.

**Lessons:**

Rupture of graft artery should be foreseen when donor-derived CRKP infection was confirmed and broad-spectrum antibiotics and other interventions need to be considered.

## 1 Introduction

*Klebsiella pneumoniae* (KP) is a Gram-negative bacilli exists in upper respiratory tract and intestine, it is known as an important opportunistic pathogen and a common cause of hospital-acquired infection.^[1]^ The drug-resistance is mainly associated with the carbapemenase in KP, as a result, a range of broad-spectrum antibiotics are ineffective to carbapenem-resistant *K pneumoniae* (CRKP), and the emergence of CRKP poses a serious threat to public health worldwide.^[1,2]^

Cadaveric solid organ transplantation (SOT) is at high stake of developing infections mainly because of the acceptance of marginal donor (with active infection), major surgical intervention and immunosuppressive agent.^[3]^ During the past 20 years, the infections in solid organ recipients associated with bacteremia have increasing significantly meanwhile there is a clear shift that the gram-negative bacteria had become the predominant pathogen,^[4]^ in which, CRKP infections are emerging as a severe complication early after SOT due to the lack of efficient antimicrobial prophylaxis.

The common infection sites of CRKP are pneumonia, urinary tract infection and intra-abdominal infection,^[5]^ little is known about the vascular complication particularly artery rupture caused by CRKP infection.

We report 3 cases of graft artery rupture due to CRKP infections derived from the same donor, in which 2 kidney recipients underwent graft nephrectomy and 1 liver recipient developed hepatic artery thrombosis and complicated intra-abdominal infection. Patients have provided informed consent for publication of the case. Moreover, we conduct a review of literature about donor-derived infection (DDI) duo to CRKP.

## 2 Cases presentation and literature review

### 2.1 Donor

The donor was a 30-year-old woman with a relapse of brain glioma 4 years after surgery, the patient was admitted to the neurosurgical intensive unit because of loss of consciousness, the endotracheal secretion, blood and urine specimen was drawn for culture and yielded negative results before transplantation. 5 days after admission, brain death was confirmed following criteria and practical guidance for determination of brain death in adults (2015). Her parents agreed to organ donation, the liver and 2 kidneys were accepted for transplantation. The donation, allocation, and reception of organs were under supervision of local ethics committee. Twenty hours after transplantation, the culture of blood from donor's portal vein (PV) was proved to be positive for CRKP.

### 2.2 Recipient 1

**Figure 1:**
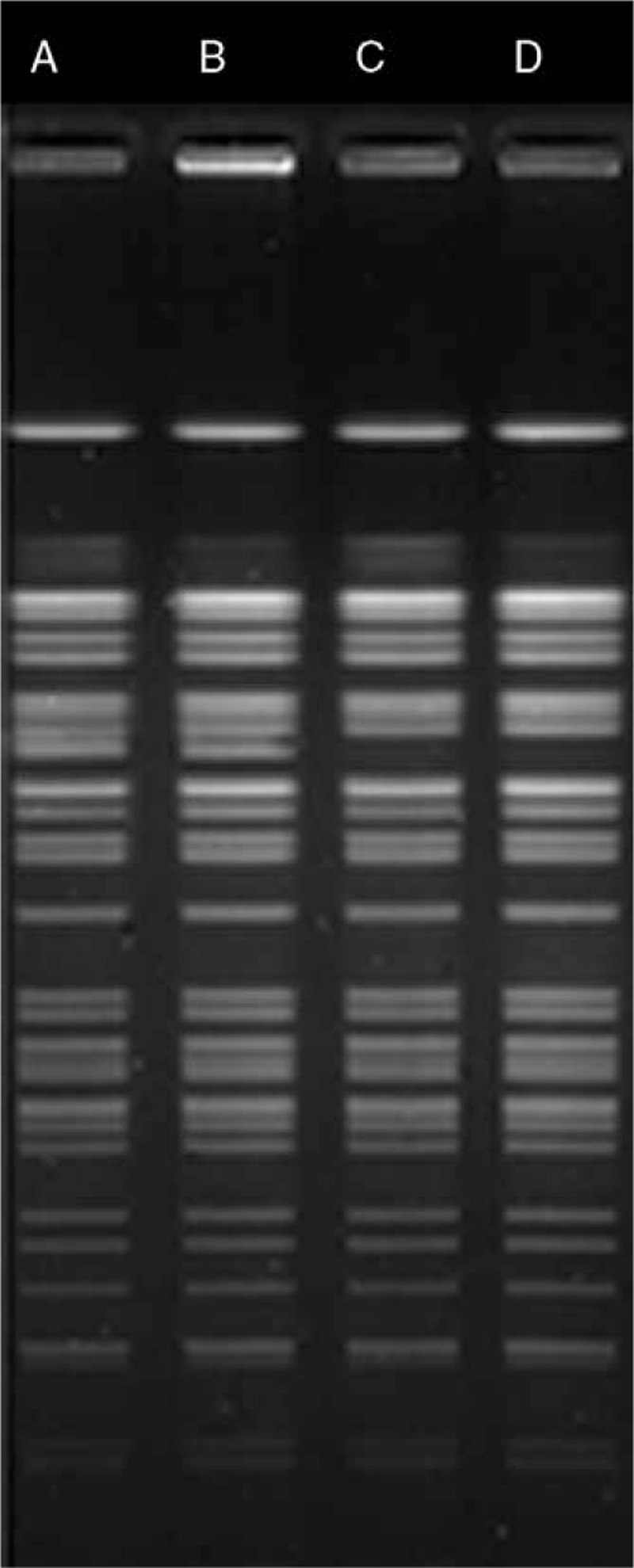
Pulsed-field gel electrophoresis analysis of total DNA of CRKP from donor and 3 recipients isolates. A, B, C, D: restriction patterns are from CRKP isolates from culture of PV blood from donor (A), liver abscess from recipient 1 (B), peripheral blood from recipient 2 (C), and surgical site sample from recipient 3 (D). CRKP = carbapenem-resistant *Klebsiella pneumoniae*.

**Figure 2:**
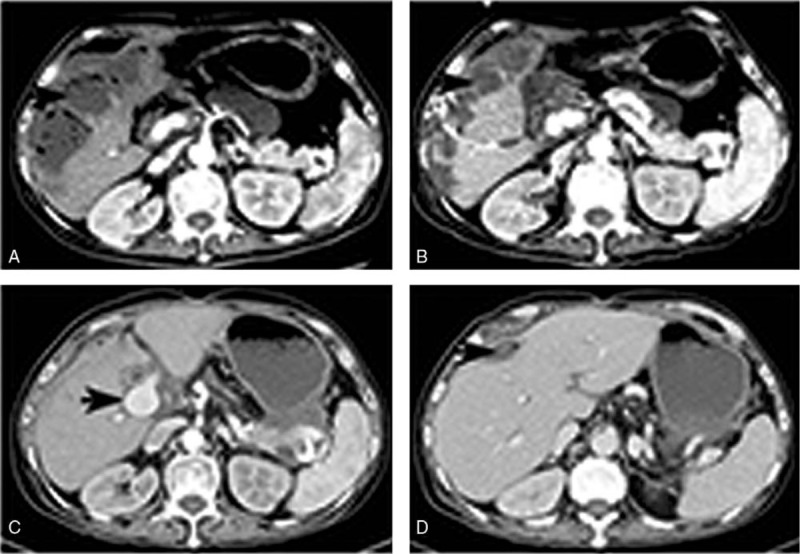
liver recipient (recipient 1). (A) Enhanced total abdomen CT scan revealed the thrombosis of hepatic artery and massive liver necrosis 14 days after liver transplantation. (B) Reduction in sizes of liver abscesses after percutaneous peritoneal drainage 2 months following liver transplantation. (C) Significant reduction in sizes of liver abscess 7 months after liver transplantation. (D) Enlarged portal vein and numerous branches 7 months after liver transplantation. CT = computed tomography.

A 55-year-old man underwent liver transplantation for recurrence of hepatocellular carcinoma. An orthotopic liver transplantation was performed using the piggyback technique without extracorporeal venovenous bypass. The cold ischemia time was 3 minutes while the warm ischemia time was 1.5 hours. The blood specimen from donor's inferior vena cava, PV, and the preservation fluid (University of Wisconsin solution) from perfused liver graft were collected for bacterial and fungal culture. A standard antimicrobial prophylaxis (Imipenem/Cilastatin 1 g every 8 hours IV + Teicoplanin 0.2 g/d IV + Micafungin 50 mg/d IV) in our center was administered. Twenty hours after surgery, the culture of blood from donor's PV and preservation fluid was found to be positive for CRKP, and the antibiotic treatment was empirically shifted to Tigecycline (50 mg/12 h IV), Meropenem (1 g/8 h IV), and Micafungin (50 mg/d IV). The recipient discharged from the intensive care unit 7 days after surgery without sign of infection. However, the patient experienced a massive intra-abdominal hemorrhage (1000 mL) and showed hemodynamic instability on the 12th day after transplantation, an emergency laparotomy was performed and the position of hemorrhage turned up to be a branch of hepatic artery, beside the branch a small abscess was found. Ligation of bleeding artery and clearance of abscess were completed meanwhile the antibiotic regimen was switched to Tigecycline (50 mg/12 h IV), Meropenem (1 g/8 h IV), and Voriconazole (200 mg/12 h IV) in consideration of fungal infection. However, the culture and susceptibility test of the abscess specimen revealed a CRKP infection which was resistant to all tested antimicrobials, except for sulfamethoxazole (SMZ). Pulsed-field gel electrophoresis (PFGE) analysis confirmed the 2 CRKP isolates from donor and recipient 100% matched in restriction pattern profile (Fig. 1). Therefore, we diagnosed the patient with donor-derived CRKP infection, the antibiotic treatment changed to polymyxin B (500000 U/d IV), SMZ (0.8 g/12 h oral), and Voriconazole (200 mg/12 h IV). Two days after reoperation, a sudden rebleeding in the abdominal cavity emerged, as a result the recipient experienced severe hemorrhagic shock. Enhanced computed tomography (CT) scan revealed a complete thrombosis of hepatic artery and massive hepatic necrosis (Fig. 2A). The rupture of arterial anastomosis was taken into consideration, which results in acute thrombosis of hepatic artery. After massive transfusion and fluid infusion, the bleeding was controlled. The patient exhibited frequent high fever, poor appetite, and general malaise, CT scan showed multiple liver and peritoneal abscesses; therefore, multiple times of percutaneous peritoneal drainage (Fig. 2B) were performed. After a 7-months course treatment of polymyxin, drainage, and enteral nutritional support, the patient's inflammatory marker levels returned to normal, and there was a significant reduction in sizes of the liver and peritoneal abscesses (Fig. 2C and D) nonetheless the culture of drainage fluid for CRKP was still positive.

### 2.3 Recipient 2

**Figure 3:**
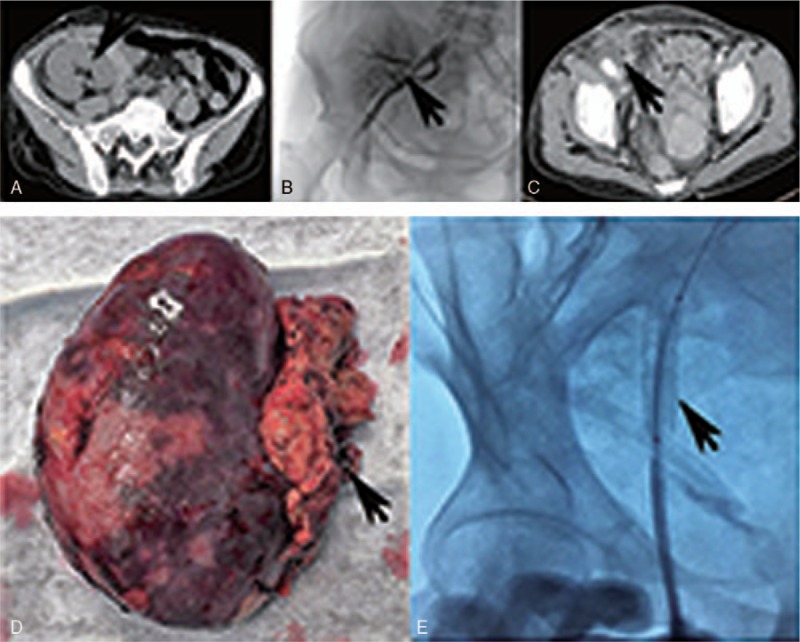
Two renal recipients (recipient 2, 3). (A) CT scan showed massive fluid collection in the perigraft area 14 days after renal transplantation in recipient 2. (B) Iliac artery angiopathy found the bleeding of renal artery anastomosis 14 days after renal transplantation in recipient 2. (C) Enhanced CT scan revealed the right iliac abscess 75 days after resection of renal allograft in recipient 2. (D) Resection renal allograft with corroded artery end 13 days after renal transplantation in recipient 3. (E) Endovascular covered stent placed in the left external iliac artery 13 days after transplantation in recipient 3. CT = computed tomography.

The recipient was a 65-year old woman underwent renal transplantation on the same day of the liver transplantation, receiving the right kidney of the same donor. The total length of operation was 3 hours and estimated blood loss was 200 mL, the antibiotic prophylaxis was Sulperazone (2 g/12 h IV), Teicoplanin (0.2 g/d IV), and Voriconazole (200 mg/12 h IV). After an uneventful postoperative course, she presented with renal graft area pain and a newly formed perigraft collection on the 14th day after surgery, the recipient then progressed rapidly into hemorrhagic shock. CT scan showed massive fluid collection in the perigraft area (Fig. 3A). Immediate right iliac artery angiography revealed the rupture of renal artery anastomosis (Fig. 3B), an endovascular covered stent was placed in the right external iliac artery to stop continually bleeding. After angiopathy, an emergency graft nephrectomy was completed, the culture of blood and perigraft fluid collection obtained at surgery were positive for CRKP, the susceptibility test showed the same result as the donor. The antibiotic regimen switched to Tigecycline (50 mg/12 h IV), Meropenem (1 g/8 h IV), and Voriconazole (200 mg/12 h IV). Five months after renal transplantation, the patient was on regular out-patient follow-up for treating the right iliac abscess (Fig. 3C) and receiving hemodialysis treatment pending a second transplantation.

### 2.4 Recipient 3

The recipient was a 30-year-old young man receiving the left kidney of the same donor, 13 days after renal transplantation, he presented left loin pain and unstable vital signs. An instant exploration found the root of renal artery ruptured, the renal graft was removed (Fig. 3D) and a sequential endovascular graft exclusion of left external iliac artery was completed (Fig. 3E). The bacteriological evaluation of the left renal graft found CRKP infection, in the meantime the PFGE analysis confirmed the homology of the CRKP strains from recipient and donor. The recipient discharged 90 days after transplantation and waited for a second transplantation while receiving hemodialysis.

### 2.5 Literature review

We implemented a Medline search using the terms “donor derived,” “donor transmitted,” and “donor colonized” combined with the terms “*Klebsiella Pneumoniae*,” “Carbapenem-Resistant *Klebsiella Pneumoniae*,” “CRKP,” “extended-spectrum beta-lactamase” and “transplantation.” Nine related published reports during 2011 to 2018 were found, involved 18 patients with donor-derived CRKP infection, consisted of 6 liver recipients, 5 lung recipients, 4 kidney recipients, 1 heart recipient, 1 combined kidney-pancreas recipient, and 1 combined liver-kidney recipient. Among which there were 1 early death (9 days)^[6]^ and 5 late death (≥28 days) postoperation, 1 lost of follow-up after renal allograft loss.^[7]^ Eleven cases of restriction pattern of isolates from donors and recipients were matched confirmed by PFGE analysis while the other 7 cases were highly suspected due to the matched antibiotic susceptibility tests. Only 1 case reported a renal allograft loss due to rupture of renal artery as a deadly complication of donor-derived CRKP infection.^[7]^ All the 18 cases of donor-transmitted CRKP infections are summarized in Table 1.^[6–14]^

**Table 1:**
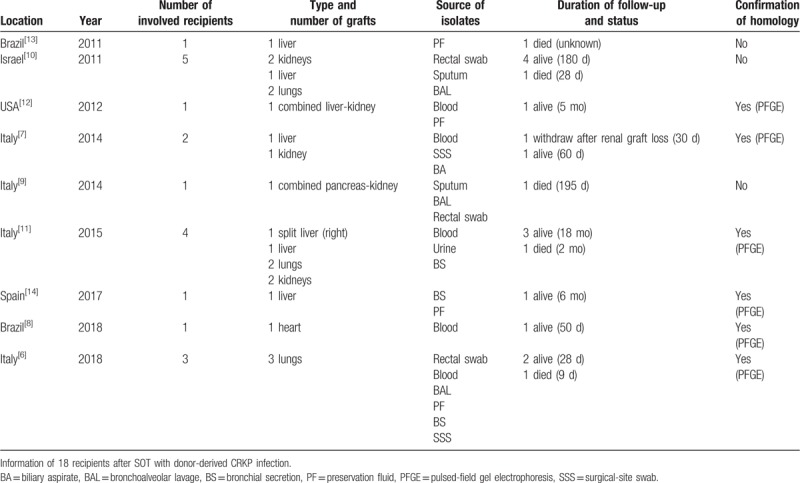
Summary of 9 reported literatures.

## 3 Discussion and conclusion

In recent years, with the continuous improvement and development of ethics, law, and public's awareness of organ donation, the SOT from donation after cardiac death (DCD) has been extensively promoted in China. Along with the prosperity of organ transplantation in China, infectious complications associated with donors become one of major problems after SOT, among which, CRKP, characterized by highly virulent, highly transmissible, and high mortality, has emerged as the most lethal type of pathogen following SOT.

Graft vascular rupture or thrombosis are occasionally found in fungal infection.^[15,16]^ As the evident shift of pathogens in SOT recipients from gram-positive bacteria and fungus to Gram-negative bacilli,^[5]^ vascular complications related to bacteremia are occasionally reported. Orlando et al^[17]^ reported a case of early renal graft loss and recipient death due to arterial anastomosis rupture caused by *Pseudomonas aeruginosa* infection, in the 5 cases presented by Wang et al,^[18]^ 4 kidney recipients experienced renal artery rupture and the other 1 was complicated with thrombosis of graft artery. However, all 6 cases mentioned above were not DDI, which is apparently different in diagnose and treatment.

In our report, 3 recipients were diagnosed with donor-transmitted CRKP infection, although antibiotic treatment was enhanced once the preservation fluid isolate was proved to be CRKP positive, lethal graft artery rupture occurred in all 3 recipients, causing 2 kidney allografts losses and 1 complicated intra-abdominal infection, accompanied by huge economic losses (approximate 300,000 USD). The infection of the liver recipient was eventually controlled by the combination of IV polymyxin B, multiple percutaneous drainages, and long-term nasal feeding enteral nutrition. The 2 renal recipients were still waiting for a second transplantation.

To our knowledge, this is the first time the high vascular corrosivity (HVC) of donor-derived CRKP which caused fatal arterial ruptures of all 3 recipients was reported. Compared with urinary tract infection, pneumonia, surgical-site or abdominal infection, vessel rupture is a rare but urgent scenario associated with noticeable higher mortality and worse outcome.

DDI is often not revealed before transplantation due to the slow growth of pathogens in vitro, furthermore, the negative pre-transplant culture of donor's specimen does not exclude preservation fluid contamination by CRKP.^[19]^ Some authors^[20]^ support the use of grafts from the DCD donors infected with CRKP under appropriate antimicrobial therapy, yet we suggest that the organ should not be implemented with the evidence of HVC-CRKP infection of donor.

Once the evidence of HVC-CRKP infection has been confirmed, antimicrobial prophylaxis contains Polymyxin B or Tigecycline should be applicated. Renal allograft nephrectomy is the final solution for graft artery rupture. For liver allograft, despite the dual blood supply from hepatic artery and PV, necrosis and abscess of liver parenchyma are inevitable. With our experience, adequate enteral nutrition, together with effective antibiotic treatment and valid abscess drainage are beneficial for recipient.

In conclusion, donor-derived CRKP infection is a great threat to solid organ recipients and health care institutions. Effective and timely communication between laboratory and clinician is crucial to control the spread of pathogen, rapid detections, and efficient antibiotics regimens remain to be further investigated.

## Acknowledgment

The authors thank the National Natural Science Foundation of China (Grant No. 81702408) for the support.

## Author contributions

**Conceptualization:** Yunshi Cai, Heng Xiao, Cheng-You Du.

**Data curation:** Yunshi Cai, Heng Xiao, Zheng-Rong Shi, Cheng-You Du.

**Formal analysis:** Shu Zhang.

**Funding acquisition:** Heng Xiao.

**Investigation:** Yunshi Cai, Heng Xiao, Shu Zhang, Mao Li, Si-Min Liang, Zheng-Rong Shi.

**Methodology:** Shu Zhang, Zheng-Rong Shi.

**Resources:** Shu Zhang, Mao Li, Si-Min Liang, Zheng-Rong Shi, Cheng-You Du.

**Validation:** Yunshi Cai, Heng Xiao.

**Visualization:** Yunshi Cai, Heng Xiao.

**Writing – original draft:** Yunshi Cai, Heng Xiao.

**Writing – review and editing:** Yunshi Cai, Heng Xiao, Zheng-Rong Shi, Cheng-You Du.

Yunshi Cai orcid: 0000-0003-1360-3217.
